# Supplementary material for: High-frequency PCR-testing as a powerful approach for SARS-CoV-2 surveillance in the field of critical infrastructure: A longitudinal, retrospective study in a German tertiary care hospital
Source: PLoS One. 2025 Feb 6;20(2):e0317009. doi: 10.1371/journal.pone.0317009 (PMC11801583; doi:10.1371/journal.pone.0317009)
Supplement: Attachment — Submitted filename: Response to reviewers.docx [file pone.0317009.s007.docx]

Response to reviewers

Additional Editor Comments:

The paper is well written and clearly presented.

Major observations:
- In Methods section Authors should describe how data was presented and how analysis was conducted;

Thanks, we added the information, that we used GraphPad Prism 10.1.2 for the visualization and statistical analysis of the data.

- About reinfections, Authors should report the incidence and in discussion section Authors should compare this result with published literature;

Thanks for this valuable comment. We reported the incidence within the “result” section (Fig. 4) and already compared our values with published literature. Based on your comment, we now calculated two different reinfection-incidences and supplemented Fig. 4 in this regard: the incidence per tested individual (Fig. 4, left Y-axis) and the incidence per total employees (Fig. 4, right Y-axis). Due to the low rate of secondary- and third reinfections, the latter was only considered for the “1st reinfection cohort”.

- Authors should also discuss how vaccination campaign impacted the surveillance.

We have now discussed this aspect in the last section of the discussion.

- I suggest also to discuss the vaccination efficacy
We have now also discussed this aspect in the last section of the discussion.

[Note: HTML markup is below. Please do not edit.]

Reviewers' comments:

Reviewer's Responses to Questions

5. Review Comments to the Author

Please use the space provided to explain your answers to the questions above. You may also include additional comments for the author, including concerns about dual publication, research ethics, or publication ethics. (Please upload your review as an attachment if it exceeds 20,000 characters)

Reviewer #1: Overall, I believe that this is a well conducted study and a well written manuscript, which deserves to be published in the peer-reviewed evidence-based literature. As such, I have recommended that it be published with a couple of minor revisions.

I would greatly appreciate if the authors could please address the following concerns:

1. Most importantly, unless I am mistaken, I believe that your conclusion statement should read as "High-frequency PCR-testing is a powerful tool concerning SARS-CoV-2 surveillance in the field of critical infrastructure. In addition, our data show that SARS-CoV-2 RNA is detectable for longer in elderly individuals compared to younger ones" as opposed to "...for longer in younger individuals compared to elderly." After all, Figure 6 clearly shows that older individuals had lower CT values (higher viral loads) for a greater number of days than younger ones, and this phenomenon is also mentioned in your Discussion section.

Thank you very much for this important suggestion. We have changed the sentence.

2. Please fix the following minor grammatical error and clarify the following:
-"high-frequency PCR-testing" in place of "high-frequent PCR-testing" - in the Abstract section.
-"November 18, 2020" in place of "November 18th" for timing of when testing became mandatory - in the Results section.

Thank you for your comments, we have made the changes.

Reviewer #2: The manuscript is meticulously crafted and well-organized, drawing from a sizable dataset of approximately 285,000 oral swab samples collected from 3,421 healthcare workers. It explores the impact of frequent PCR testing for SARS-CoV-2 surveillance during the COVID-19 pandemic. The statistical methods employed are robust and thoroughly documented, ensuring the reliability of the findings. The manuscript effectively communicates complex findings in an accessible manner, providing clear explanations for the study's choices and enhancing the overall clarity of the presentation.

The manuscript effectively utilizes a large dataset to address a critical issue in healthcare. While it is statistically robust and well-written, its retrospective design and setting-specific conclusions may limit its broader applicability. A more thorough discussion of potential biases, including the impact of external factors (e.g., changes in public health policies or vaccine uptake) on the results, would strengthen the manuscript.

Thanks a lot for your advice. We have expanded the discussion to include these aspects.

The study cites 3,421 healthcare workers, while the Heart and Diabetes Center (HDZ) has around 2,500 employees. Could you clarify if the study included additional staff members beyond the healthcare workers, or if there might be another explanation for the difference in these figures?

Thanks for this important demand. The general number of employees at our hospital is around 2,500. The difference between individuals screened by PCR and employees is explainable by a regular staff turnover of employees per month accumulating over the 2.5 year period. We added this aspect to the manuscript.
